# Supplementary material for: Chromatin interactions and expression quantitative trait loci reveal genetic drivers of multimorbidities
Source: Nat Commun. 2018 Dec 5;9:5198. doi: 10.1038/s41467-018-07692-y (PMC6281603; doi:10.1038/s41467-018-07692-y)
Supplement: Supplementary file 1 — SupplementaryInformation [file 41467_2018_7692_MOESM1_ESM.pdf]

Chromatin interactions and expression quantitative trait loci reveal genetic drivers of multimorbidities

Fadason et al

Supplementary Information

Supplementary Figures

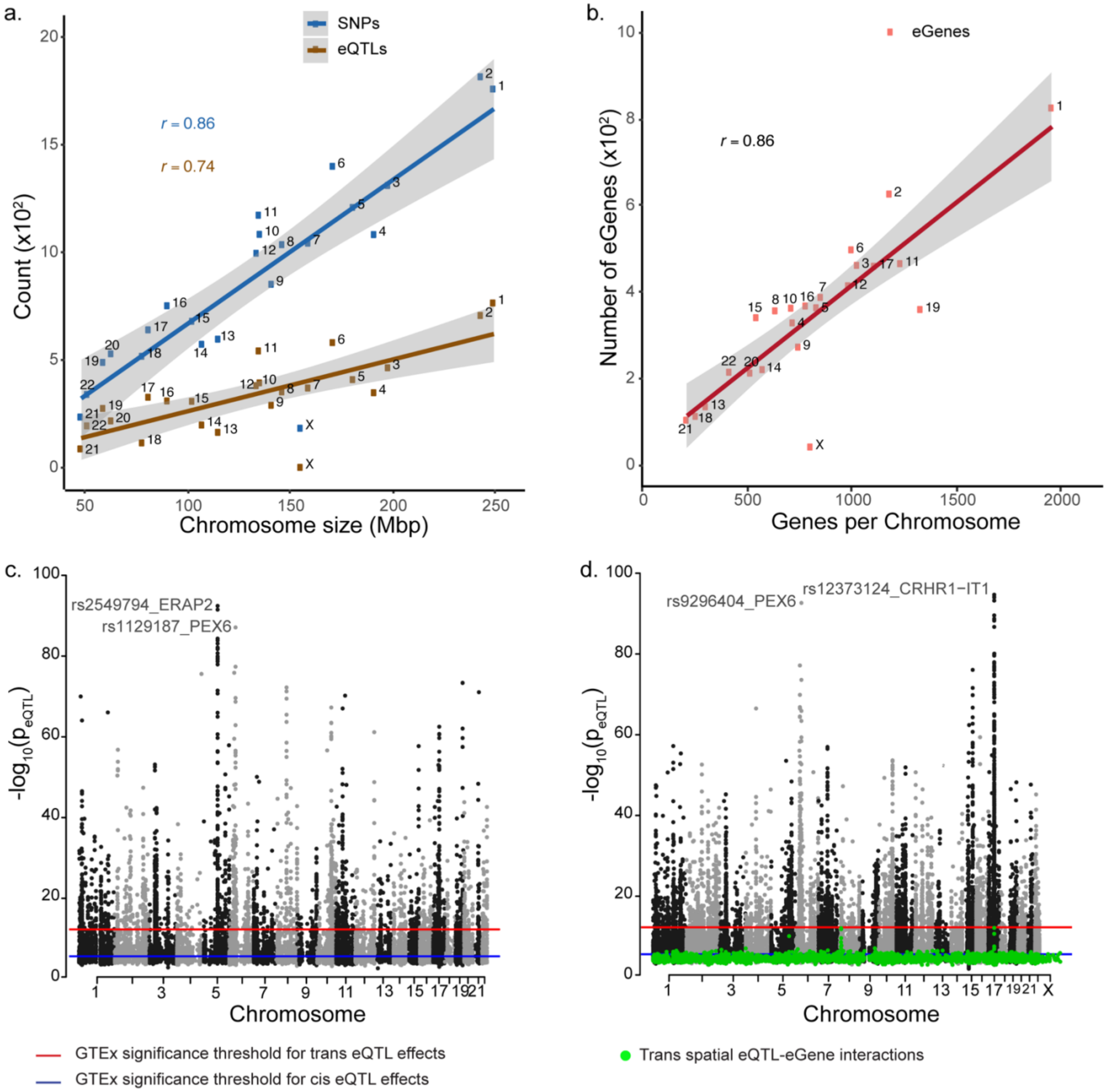

Supplementary Figure 1. **Summary of eQTL-eGene interactions.**

(a) Correlation between chromosome size with number of SNPs (*n* = 20,782) and eQTLs (*n* = 7,776). (b) Correlation between number of eGenes on chromosomes and the total number of genes on chromosomes. (c) Manhattan plot showing the eQTL p-values (GTEx v4) of significant (*FDR* ≤ 0.05) GWAS-mapped eQTL-eGene interactions in tissues. (d) Manhattan plot of significant eQTL-eGene interactions that are not mapped in the GWAS Catalog. We considered an interaction as novel if the eGene is not distinctly mentioned in the 'MAPPED_GENE' column of a SNP association in the GWAS Catalog (v1.0.1). For c) and d); X axis tick marks are organized sequentially from 1-22, followed by the X chromosome. The Y chromosome is not represented on c) or d).

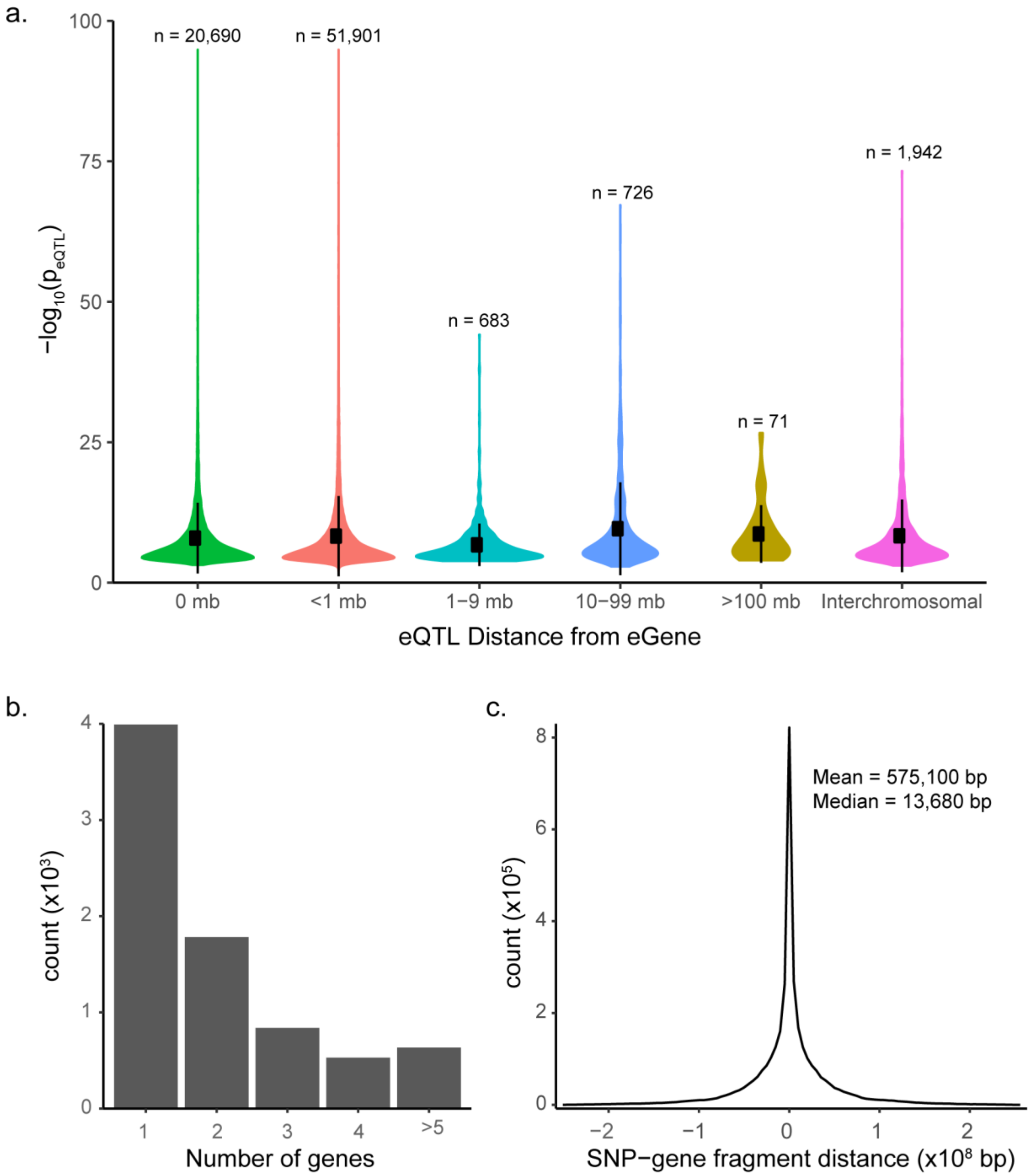

Supplementary Figure 2. **Relationships between eQTL SNPs and eGene.**
(a) Violin plot of eQTL p-values and distance between eQTL SNPs and eGene shows that there are more eQTLs within 1 mb of genes than there are within genes. (b) About half of eQTL SNPs affect only one gene, the other half affect multiple genes. (c) Distribution of distance between eQTL SNP and eGene Hi-C fragment loops. The distances here are between the closest SNPs and genes.

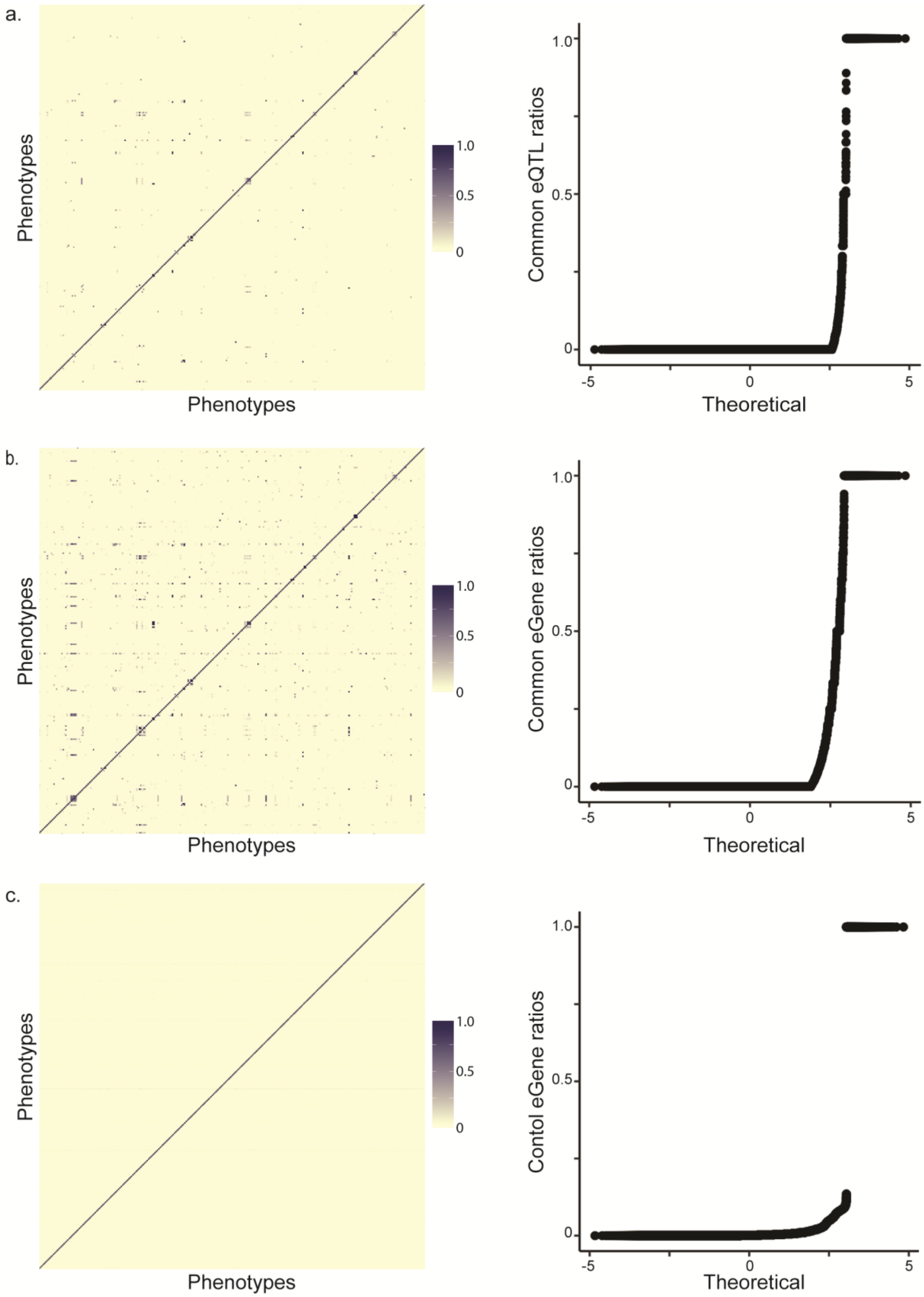

Supplementary Figure 3. **Associations of phenotypes based on shared spatial interactions.**
(a) Phenotypes associate weakly when the 7,776 significant eQTLs were used to define their interrelationship as shown by sparser blue dots in the heatmap and gaps on the Q-Q plot. (b) Relationships among phenotypes are enhanced by the eGenes. (c) 1000 null datasets were generated by randomly assigning eGenes to phenotypes such that each control phenotype has the same number as the corresponding sample phenotype. The mean null dataset has a different pattern from that of sample phenotypes. Heatmaps in a, b and c are extracts of the same set of phenotypes in the same order. Darker squares in matrices indicate higher proportions of shared eGenes (with 1 being the highest, meaning the sets of eGenes of two phenotypes are the same). Q-Q plots include shared eQTL, eGene or control ratios for 861 phenotypes.

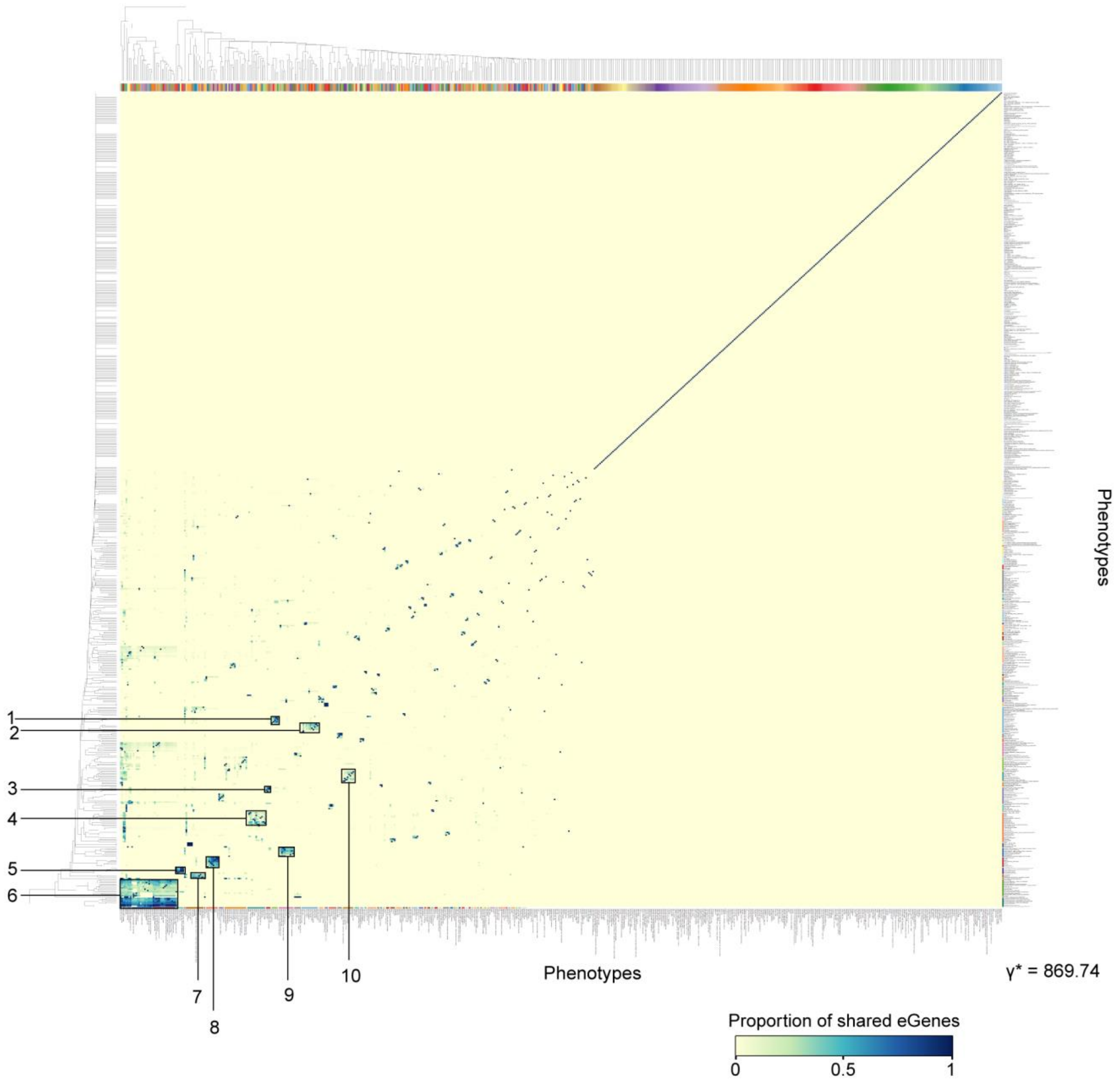

Examples of multimorbid clusters

1: Hypertension, systolic and diastolic blood pressure, mean arterial pressure, pulse pressure
2: Ovarian cancers, Alzheimers, interstitial lung disease, Parkinson's disease, progressive supranuclear palsy
3: Large artery stroke, coronary heart disease, myocardial infarction, white matter hypersensitivity
4: Allergy, neutrophil count, asthma, type 1 diabetes, rheumatoid arthritis, cervical carcinoma, biliary liver cirrhosis
5: Iron biomarkers, autism spectrum disorder, transferrin
6: PUFAS, IBD, Crohn's disease, cholesterols, height, laryngeal squamous carcinoma, muscle strength
7: Mood disorder, BMI, bipolar, schizophrenia, open-angle glaucoma, osteoarthritis, depression, adiponectin, ADHD
8: Melanoma, skin and facial pigmentation, basal cell carcinoma, freckles, suntan, sunburn
9: COPD, lung carcinoma, smoking behavior, emphysema, chronic bonchitis, lung carcinoma
10: Vitamin B12, eye measurement, carcinoembryonic antigen, atopic eczema, psoriasis, endothelial growth factor

Supplementary Figure 4. **Phenotypes cluster based on common spatial eGenes.**
The heat map highlights the multimorbidity of 618 phenotypes that share ≥ 4 spatial eGenes with at least one other phenotype. Unsupervised clustering was performed using convex biclustering algorithm from the cvxbiclustr R package. Deep blue squares indicate higher proportions of shared eGenes, with 1 being the highest and indicating that two phenotypes have the same set of eGenes. Ten notable clusters are annotated and described. A complete list of inter-phenotype shared eGene proportions is presented in Supplementary Data 2. To see phenotypes in these clusters, a high resolution version of this image is available at DOI 10.17608/k6.auckland.7294934

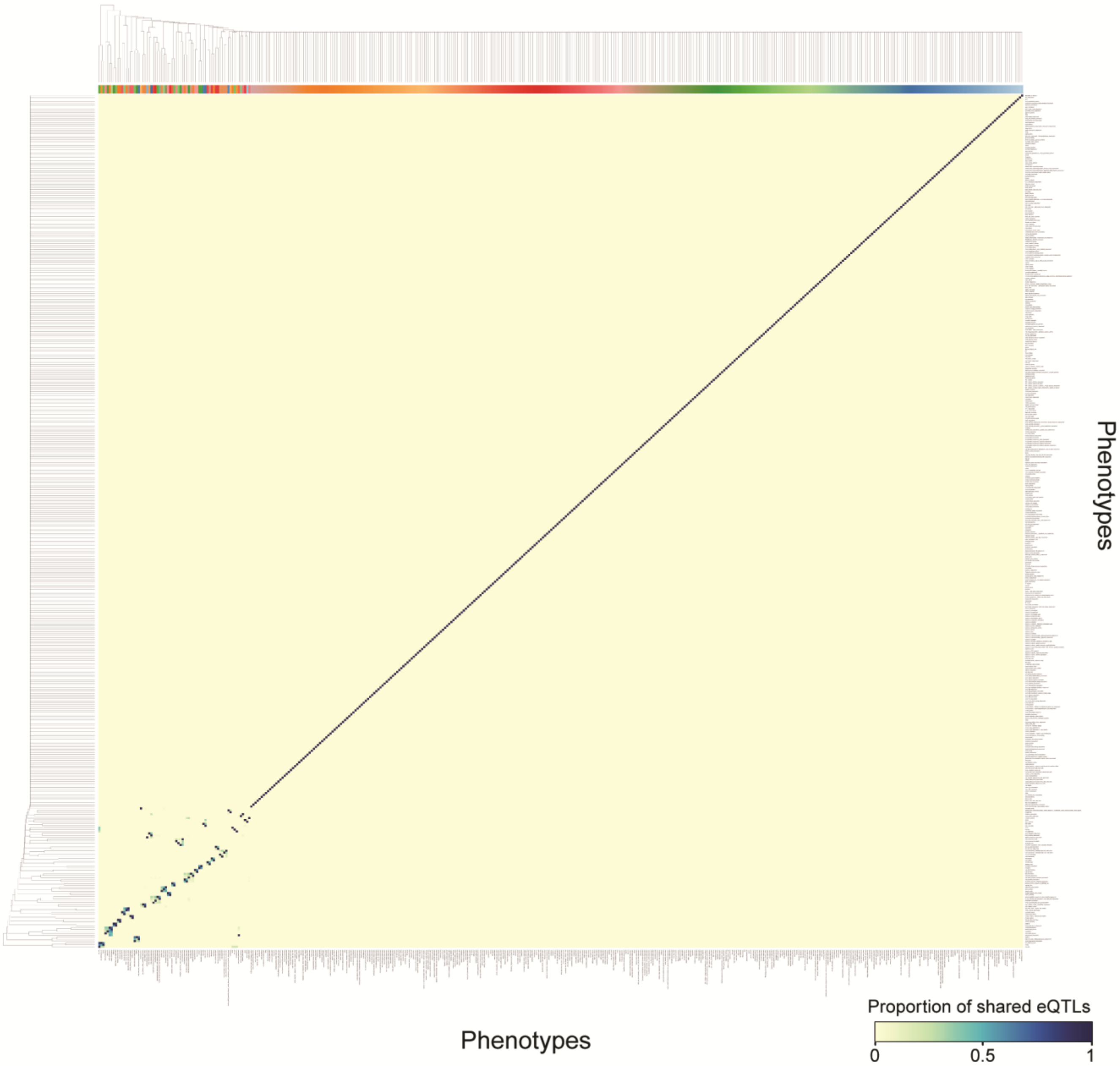

Supplementary Figure 5. **Biclustering of phenotypes cluster based on the shared eQTLs.**
The graph shows the segregation of phenotypes (on both axes) that share ≥ 4 eQTLs with at least one other phenotype by the convex biclustering algorithm from the cvxbiclustr R package. Deep blue squares indicate higher proportions of shared eGenes, with 1 being the highest and indicating that two phenotypes have the same set of eGenes. The complete proportions of the eQTLs shared among phenotypes are given in Supplementary Data 2. We designated that phenotypes must share ≥ 4 eQTLs with at least one other phenotype because the ratio of eGenes to SNPs (*i.e.* 7776 / 7917) is approximately one and this made it equivalent to the requirements for eGenes biclustering (Supplementary Figure 4).

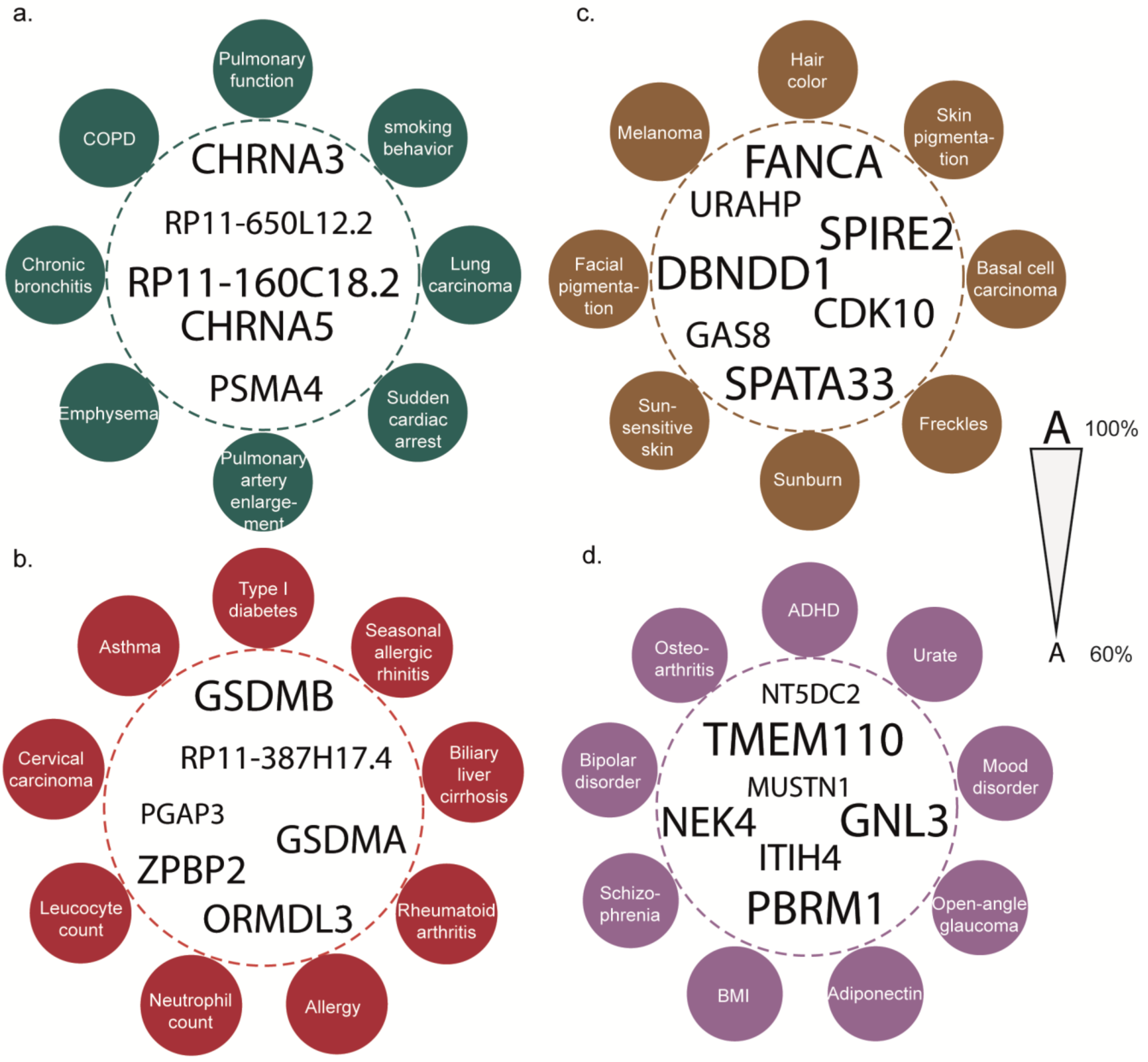

Supplementary Figure 6. **Examples of phenotype clusters.**

(a) The *CHRNA5–ADAMTS7* region (chromosome 3:78,832,747 – 79,103,773; hg19) is important for the clustering of disorders of pulmonary function: chronic obstructive pulmonary disease, smoking behaviour, lung carcinoma and sudden cardiac arrest. (b) Immune disorders such as type 1 diabetes, cervical cancer, rheumatoid arthritis, and biliary liver cirrhosis are clustered around the *PGAP3 – GSDMA* region on chromosome 17: 37,827,375 – 38,134,431. (c) Skin pigmentation, hair colour, facial pigmentation, melanoma, basal cell carcinoma, freckles and sunburn share genes in the *SPATA33 – URAHP* (chromosome 16:89724152 – 90114191) region. (d) Genes from the *NT5DC2* - *TMEM110* (chromosome 3:52558385 – 52931597) region are common to mental disorders, body mass index, osteoarthritis, open-angle glaucoma and adiponectin measurement. Proportions of eGenes for the four clusters are given in Supplementary Data 3.

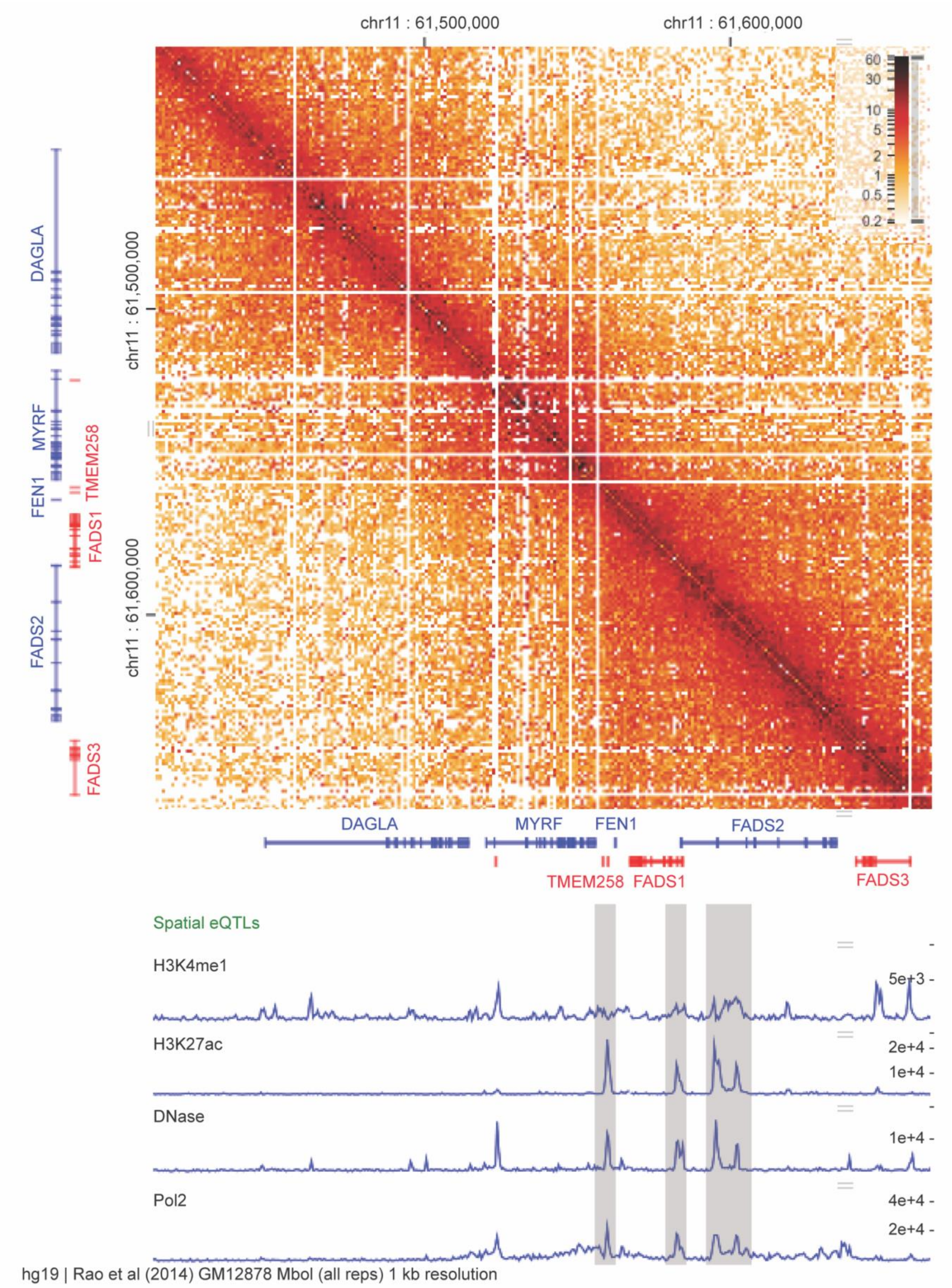

Supplementary Figure 7. **eGenes are affected distally by eQTLs.**
eQTLs in the fat metabolism cluster are found in putative enhancer regions (as marked by epigenetic markers) and have both intra- and inter-TAD effects on genes. Hi-C image was produced with HiGlass (higlass.io)

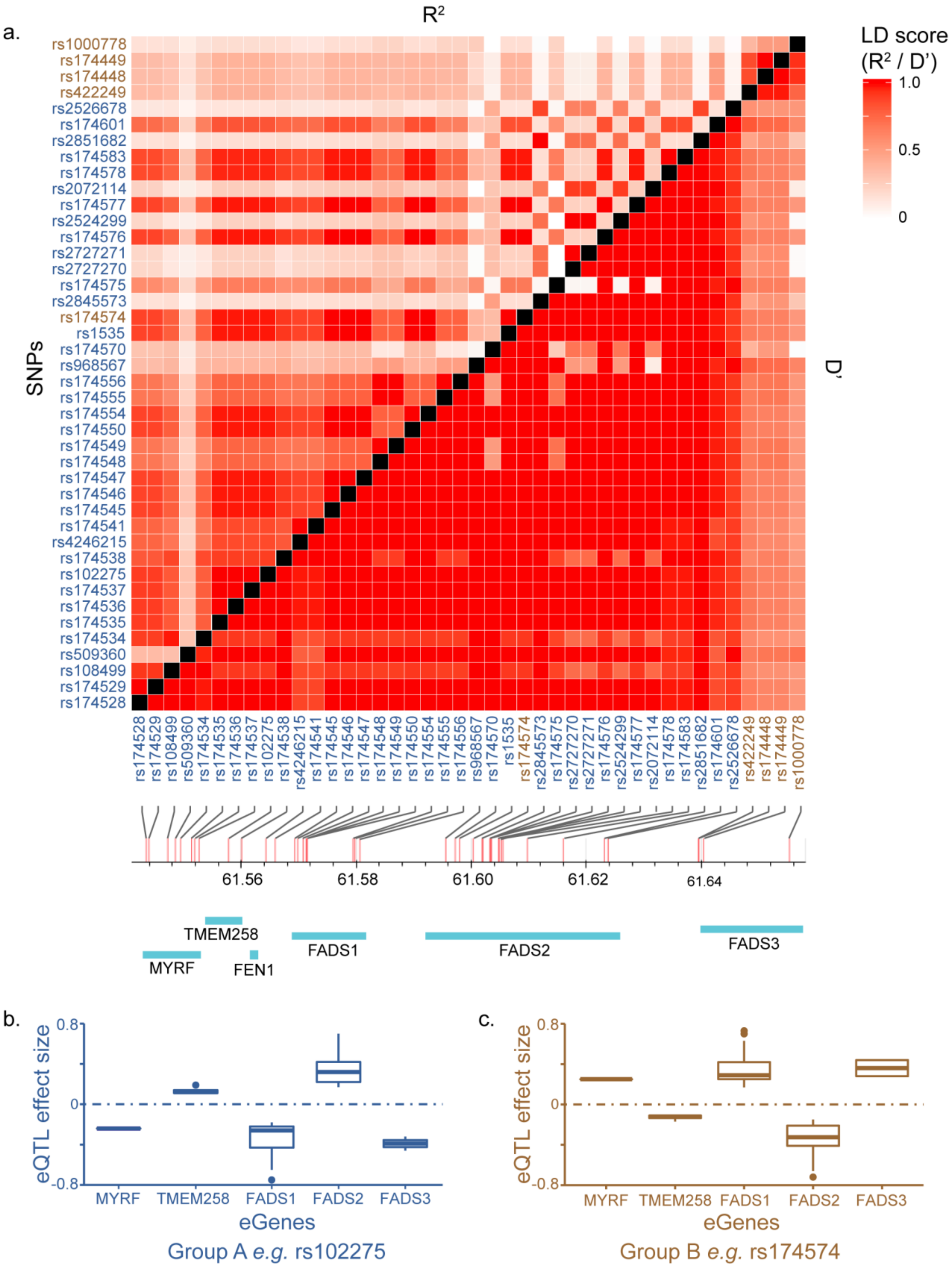

Supplementary Figure 8. eQTLs have effect sizes that are dependent on allele frequency.

(a) Linked eQTLs in the *FADS* locus have different allele specificities in the CEU population, as indicated by the differences between the D' and R^2 scores. The difference in allele frequencies is associated with the 2 distinct eQTL effect patterns (b and c) across the *FADS* locus. Groups A and B highlight the distinct pattern of eQTL associated transcriptional affects. Effect sizes of spatial eQTL on eGenes were obtained from GTEx v7 analysis. Centre line, bounds of box, and whiskers of boxplots represent the median, 2nd and 3rd quartile, and minimum and maximum values respectively.

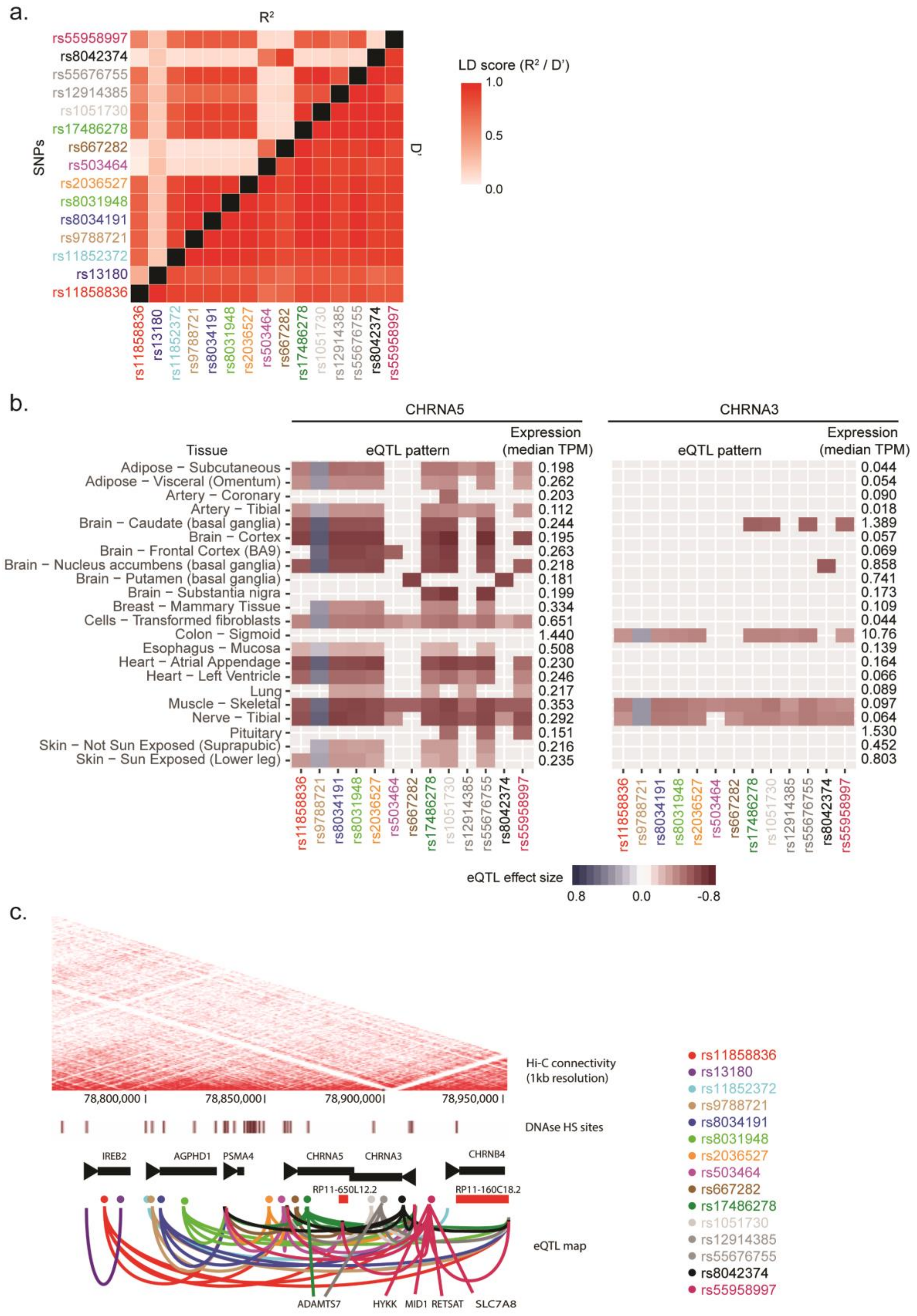

Supplementary Figure 9. **The CHRNA regulome is central to pulmonary disorders**.

(a) The LD pattern here suggests 2 alternating haplotype blocks, with the smaller block represented by rs503464. rs667282, rs8042374, and to a lesser extent, rs13180. (b) CHRNA3 and CHRNA5 are differentially expressed and regulated in the same tissues and by the same eQTLs, which seem consistent with the LD patterns in a. (c) eQTLs in the CHRNA locus region interact with multiple genes both within and outside this region, suggesting that the locus may be a super-enhancer. rs8042374, which is located at a TAD boundary also interacts with genes in the two adjacent TADs.

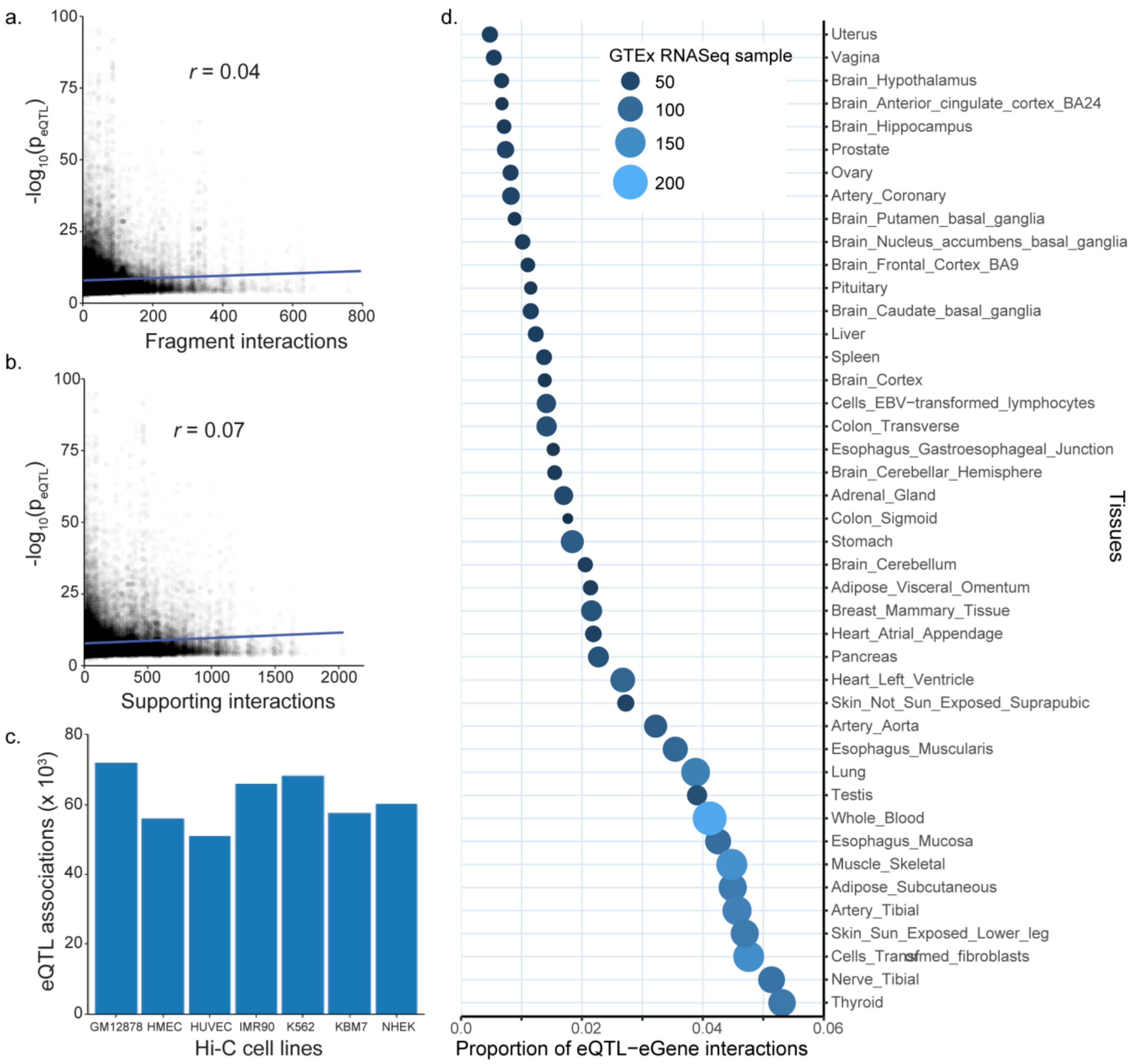

Supplementary Figure 10. **Tissue specificity of spatial eQTL-eGene interactions.**
There is no correlation between eQTL p-values and the total unique Hi-C interactions (a), and the total supporting interactions (b) of eQTL SNP-eGene associations. Interactions and supporting interactions are as defined in Fig 1a. (c) Distribution of eQTL-eGene interactions among the Hi-C cell lines. (d) The proportion of spatial eQTL-eGene associations in tissues positively correlates (r = 0.87) with the number of RNASeq and genotyped samples in GTEx.

Supplementary Tables

Supplementary Table 1. Hi-C cell lines used in this study.

Cell line	GEO IDs of replicate samples
GM12878	GSM1551552, GSM1551553, GSM1551554, GSM1551555, GSM1551556, GSM1551557, GSM1551558, GSM1551559, GSM1551560, GSM1551561, GSM1551562, GSM1551563, GSM1551564, GSM1551565, GSM1551566, GSM1551567, GSM1551568, GSM1551569, GSM1551570, GSM1551571, GSM1551572, GSM1551573, GSM1551574
HMEC	GSM1551607,GSM1551608, GSM1551609, GSM1551610, GSM1551611, GSM1551612,
HUVEC	GSM1551629, GSM1551630, GSM1551631
IMR90	GSM1551599, GSM1551600, GSM1551601, GSM1551602, GSM1551603, GSM1551604, GSM1551605
K562	GSM1551618, GSM1551619, GSM1551620, GSM1551621, GSM1551622,GSM1551623
KBM7	GSM1551624, GSM1551625, GSM1551626, GSM1551627, GSM1551628
NHEK	GSM1551614, GSM1551615, GSM1551616
