# Supplementary material for: Chromatin interactions and expression quantitative trait loci reveal genetic drivers of multimorbidities
Source: Nat Commun. 2018 Dec 5;9:5198. doi: 10.1038/s41467-018-07692-y (PMC6281603; doi:10.1038/s41467-018-07692-y)
Supplement: Supplementary file 3 — Description of Additional Supplementary Files [file 41467_2018_7692_MOESM3_ESM.pdf]

Description of Additional Supplementary Files

Supplementary Data 1. eGenes identified in GWAS traits
The eQTLs, the GWAS trait(s) with which the 7,917 eGenes are associated, and whether the eGene interaction with the eQTLs is cis, trans or both is given for each eGene.

Supplementary Data 2. eQTL associations on Chromosome 22
Disease-associated SNPs on chromosome 22 were tested for eQTL associations using GTEx analysis (v7) and the CODeS3D pipeline. This dataset contains significant associations (*FDR* ≤ 0.05) from both methods.

Supplementary Data 3. Pleiotropy matrices
The Phenotype eQTL overlap sheet has names of phenotypes in columns TraitX and TraitY. The number of eQTLs identified for TraitX and TraitY are given in eQTLsX and eQTLsY. The column, Common, is the number of eQTLsY that are in eQTLsX. Ratio is Common divided by eQTLsX. The Phenotype eGene overlap sheet has names of phenotypes in columns TraitX and TraitY. The number of eQTLs identified for TraitX and TraitY are given in eGenesX and eGenesY. The column, Common, is the number of eGenesY that are in eGenesX. Ratio is Common divided by eGenesX. In the Control eGene overlap sheet, all 7,917 eGenes were pooled together and randomly assigned to phenotypes so that each phenotype in the control matrix had the same number of eGenes as its corresponding phenotype in the eGene matrix. The pairwise ratios of common eGenes among the phenotypes were calculated as done in the eGene matrix. 1000 different null datasets were

constructed in this manner and the mean matrix was calculated. The Ratio column is the mean of 1000 null sets between phenotypes in columns TraitX and TraitY.

Supplementary Data 4. Phenotype-eGene associations in some multimorbid clusters
Convex biclustering of shared eGenes among phenotypes resulted in several clusters (Supplementary Figure 4). For each eGene in a cluster, an eGene score (*i.e.* the percentage of the phenotypes whose associated variants affect the expression of the eGene) is calculated. This data gives for each eGene in the clusters in Figure 2 and Supplementary Figure 6, the eGene score, number of traits with which the eGene is associated, and its eQTL pairs.

Supplementary Data 5. eQTLs in the poly-unsaturated fat metabolism cluster
The SNPs in the *FADS* locus involved in fat metabolism reveal two patterns of eQTL effects (Figure 4 and Supplementary Figure 8). Here, the reference and alternate alleles of the SNP positions are given with their frequencies.

Supplementary Data 6. eGenes in mendelian disorders.
Analysis of the OMIM data shows that eGenes, which are also involved in rare Mendelian disorders are spatially associated with eQTLs marked by common variants. These eGenes are given, together with the phenotypes they are associated with.
